# Exogenous lactate augments exercise-induced improvement in memory but not in hippocampal neurogenesis

**DOI:** 10.1038/s41598-023-33017-1

**Published:** 2023-04-10

**Authors:** Deunsol Hwang, Jisu Kim, Sunghwan Kyun, Inkwon Jang, Taeho Kim, Hun-Young Park, Kiwon Lim

**Affiliations:** 1 Laboratory of Exercise and Nutrition, Department of Sports Medicine and Science in Graduate School, Konkuk University, Seoul, Republic of Korea; 2 Physical Activity and Performance Institute (PAPI), Konkuk University, Seoul, Republic of Korea; 3 Department of Physical Education, Konkuk University, Seoul, Republic of Korea

**Keywords:** Adult neurogenesis, Nutrition

## Abstract

Adult hippocampal neurogenesis (AHN), the lifelong process of formation of new neurons in the mammalian brain, plays an important role in learning and memory. Exercise is an effective enhancer of AHN; however, the molecular mediators of exercise-induced AHN are unknown. Recently, lactate was considered as an important mediator of exercise-induced AHN. Therefore, we hypothesized that exercise with lactate intake could augment exercise-induced AHN. This study was conducted for 5 weeks with 7-week-old ICR male mice that performed mild-intensity exercise (just below lactate threshold, 55–60%VO_2max_) with or without oral administration of lactate 5 days/week. Cell proliferation, neuronal differentiation, neurogenesis-relevant factors, reference and retention memory, and spatial working memory were evaluated at the end of the experiment. The results showed that AHN was enhanced by lactate intake, but exercise-induced AHN was not augmented by exercise with lactate intake. Nevertheless, exercise-induced improvement in reference and retention memory was augmented by exercise with lactate intake. And spatial working memory was promoted by the co-treatment, also protein expression of hippocampal FNDC5, BDNF, PGC1α, and MCT2 were elevated by the co-treatment. Therefore, our findings suggest that lactate has a potential to be developed as a novel supplement that improves the positive effects of exercise on the hippocampus and its cognitive function.

## Introduction

Adult hippocampal neurogenesis (AHN) refers to the lifelong process of formation of new neurons in the dentate gyrus (DG) of the adult mammalian brain. It plays an important role in learning and memory; thus, it is associated with cognitive deficits in neurodegenerative conditions. An impairment of AHN has been shown to cause cognitive decline^1^. Therefore, promoting AHN is considered a substantial way to prevent or ameliorate cognitive deficits, which is important for improving the quality of life because of global acceleration of risk factors for neurodegenerative conditions, such as aging, Alzheimer’s disease (AD), obesity, and physical inactivity^2–4^.

Exercise effectively enhances AHN. Studies have shown that regular exercise rescues AHN impairment caused by aging^5^ and chronic stress^6^. Deteriorated AHN in an AD model was also ameliorated by long-term exercise^7^. In addition to these studies on rodents with neurodegenerative conditions, AHN was enhanced by endurance exercise training, even in normal adult rodents^8–10^. Although the effect of exercise on AHN is well-demonstrated, what molecule primarily regulates exercise-induced AHN and triggers neurogenesis-relevant factors such as brain derived neurotrophic factor (BDNF) and fibronectin type III domain containing 5 (FNDC5), important mediators of exercise-induced AHN^11–13^, are not yet known.

Lactate, which are known as just byproduct of glycolysis and a primary factor in fatigue during exercise, has been highlighted as a signaling molecule in the brain^14^. In mice, pharmacological disruption of lactate transport to hippocampal neurons impaired memory formation, and this impairment was fully reversed by intrahippocampal injection of lactate, but not glucose^15^. This result indicates that lactate acts as a signaling molecule rather than an energy substrate in the brain during memory formation.

Recently, lactate has been considered a primary mediator for exercise-induced AHN^16,17^ because the effect of lactate parallels the beneficial effects of exercise on the hippocampus^18,19^. Previous in vitro studies showed that lactate treatment upregulated the protein expression of BDNF in hippocampal cells^20^ and enhanced the proliferation of neural precursor cells obtained from the hippocampus of adult mice by shortening the generation time of neural precursor cells^21^. Similarly, in vivo studies using an inhibitor of lactate transporters (monocarboxylate transporters; MCTs) increased mRNA expression levels of hippocampal BDNF through exercise, but this increase was abolished by inhibition of MCT1/2 (MCT1: transporter for efflux of lactate, MCT2: transporter for influx of lactate only in neurons)^22^. Further, the inhibition of MCT1/2 impeded the beneficial effect of exercise on learning and memory. These results indicate that lactate is involved in AHN. Indeed, long-term injection of lactate significantly elevated AHN, but the elevation was blocked by co-treatment with an MCT2 inhibitor^23^, and lactate injection also enhanced the protein expression of hippocampal BDNF and FNDC5^22^ in rodents.

Collectively, these observations indicate that lactate promotes AHN and may be a primary mediator of exercise-induced AHN. Therefore, we hypothesized that exercise with lactate intake can augment exercise-induced AHN. Specifically, exogenous lactate can cross the blood–brain barrier via MCT1^24^ and subsequently elevate both the hippocampal extracellular lactate concentration^25^ and the hippocampal lactate concentration via MCT2^22^. To our knowledge, this is the first study to investigate the effect of co-treatment with exercise and lactate on AHN as well as to show the effect of lactate on AHN via oral administration.

## Results

### Blood lactate concentration was sufficiently elevated by oral administration of lactate and not by mild exercise

**Figure 1:**
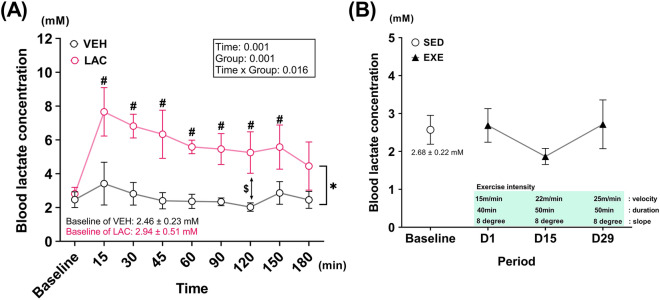
Blood lactate concentration was sufficiently elevated by oral administration of lactate and not by mild exercise. (**A**) Blood lactate concentration after oral administration of 3 g/kg lactate over time (VEH, saline administration group, *n* = 4; LAC, lactate administration group, *n* = 6). Data were analyzed using two-way repeated ANOVA with Student’s *t*-test between groups for post hoc test and paired *t*-test for comparison in baseline and a timepoint within group. (**B**) Blood lactate concentration immediately after mild-intensity exercise training over period (*n* = 8 per group; SED, sedentary group; EXE, exercise training group). For comparison within EXE, one-way repeated ANOVA was used, and comparison between SED and a timepoint of EXE was performed using independent Student’s *t*-test. D1, experimental day 1; D15, experimental day 15; D29, experimental day 29; ^#^*p* < 0.05, baseline vs time point within group; *p < 0.05, VEH vs LAC at the same time point except baseline; ^$^*p* = 0.01, VEH vs LAC analyzed by Mann–Whitney test. Data are presented as the mean ± standard deviation.

First, we conducted a pilot experiment to know whether either lactate or exercise intervention could satisfy our criterion with 6-week-old male ICR mice. Oral administration of 3 g/kg lactate significantly elevated blood lactate concentration to 7.66 ± 1.43 mM, 15 min after administration [Fig. 1A; baseline vs. 15 min in the lactate intake group (LAC): *p* = 0.001, vehicle group (VEH) vs. LAC at 15 min: *p* = 0.001]. Circulating lactate transports into the central nervous system^26,27^ via MCTs^28^, and MCTs are particularly abundant in the hippocampus compared to other regions of brain^28^. Therefore, we assert that oral administration of 3 g/kg lactate, an applied treatment, can affect the hippocampus.

We set the exercise intensity as “mild” (just below lactate threshold, 55%–60% VO_2max_)^29–31^, considering that an extremely low or high exercise intensity would be ineffective on the AHN^8,25,32^. To ensure that the exercise intensity is equally maintained throughout the entire experiment (equalization of relative exercise intensity), we had gradually increased the treadmill speed and/or exercise duration over time (increasing absolute exercise intensity; Fig. 1B), based on our previous studies^33–35^. The increase in absolute exercise intensity is required because chronic exercise enhances the exercise ability (occurrence of an exercise adaptation), and the enhanced exercise ability subsequently leads to decrease in the relative exercise intensity if the absolute exercise intensity is not increased.

In exercise training group, blood lactate levels measured immediately after exercise on experimental days 1 (D1), 15 (D15), and 29 (D29) did not differ from the baseline lactate level (Fig. 1B). Therefore, our exercise protocol was verified to have an intensity below the lactate threshold. Of note, the unchanged level of blood lactate does not mean that lactates are not produced during the exercise; this results from an increase in both production and consumption of lactate in balance when exercise intensity is set below the lactate threshold.

### Exercise with lactate administration did not augment exercise-induced AHN, but lactate administration promoted AHN

**Figure 2:**
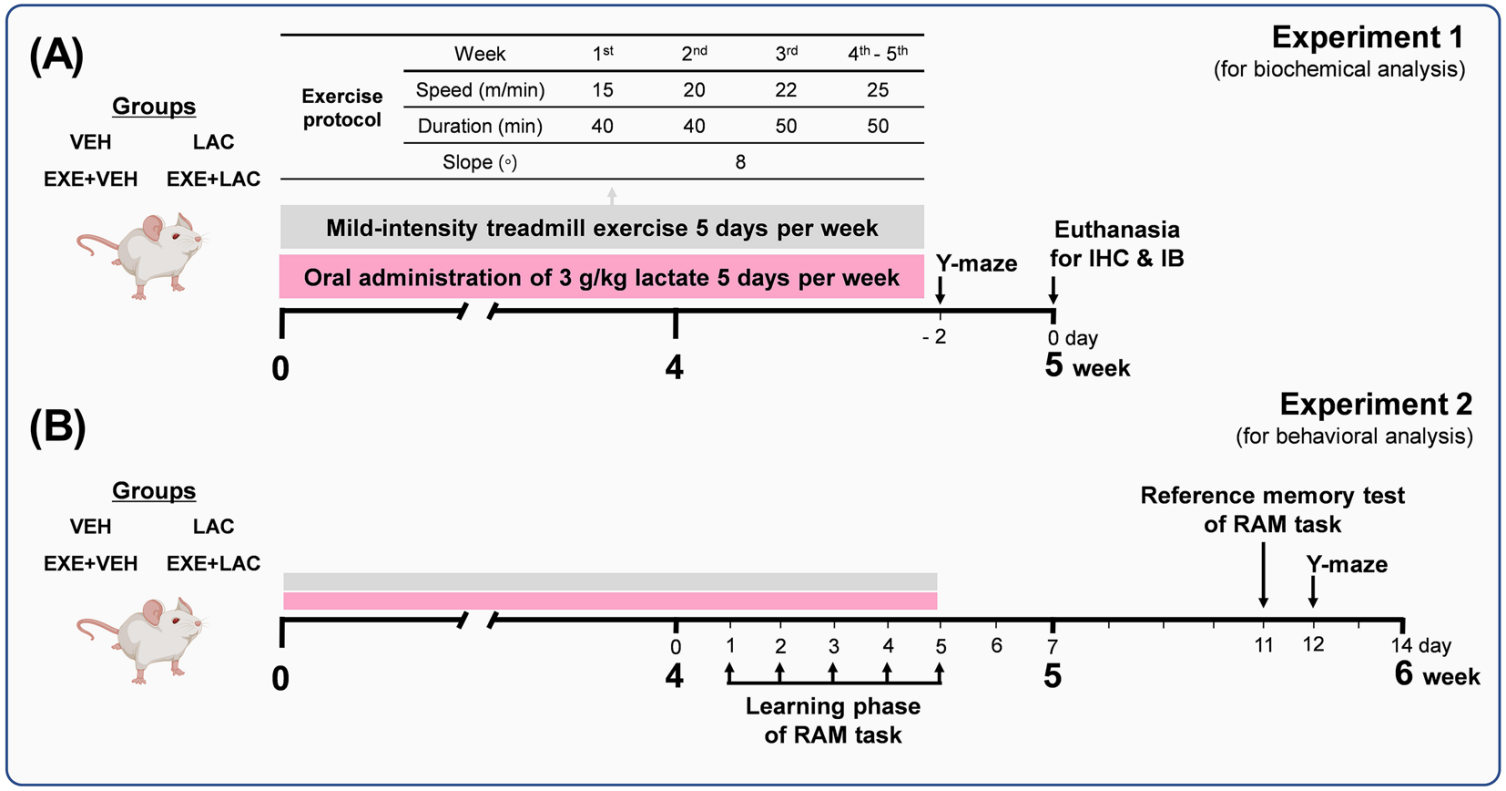
The schematic representation of experimental procedure. The experiments started with 7-week-old male ICR mice. In case of EXE + LAC, mice were administrated lactate immediately after exercise. This study comprised two independent experiments except the pilot test: (**A**) one mainly for biochemical analysis (*n* = 9 per group) and (**B**) the other for behavioral analysis (*n* = 8 per group). *VEH* sedentary without lactate, *LAC* sedentary with lactate, *EXE + VEH* exercise training without lactate, *EXE + LAC* exercise training with lactate, *IHC* immunohistochemistry, *IB* immunoblotting, *RAM* radial arm maze. The mouse icon is “Created with BioRender.com”.

**Figure 3:**
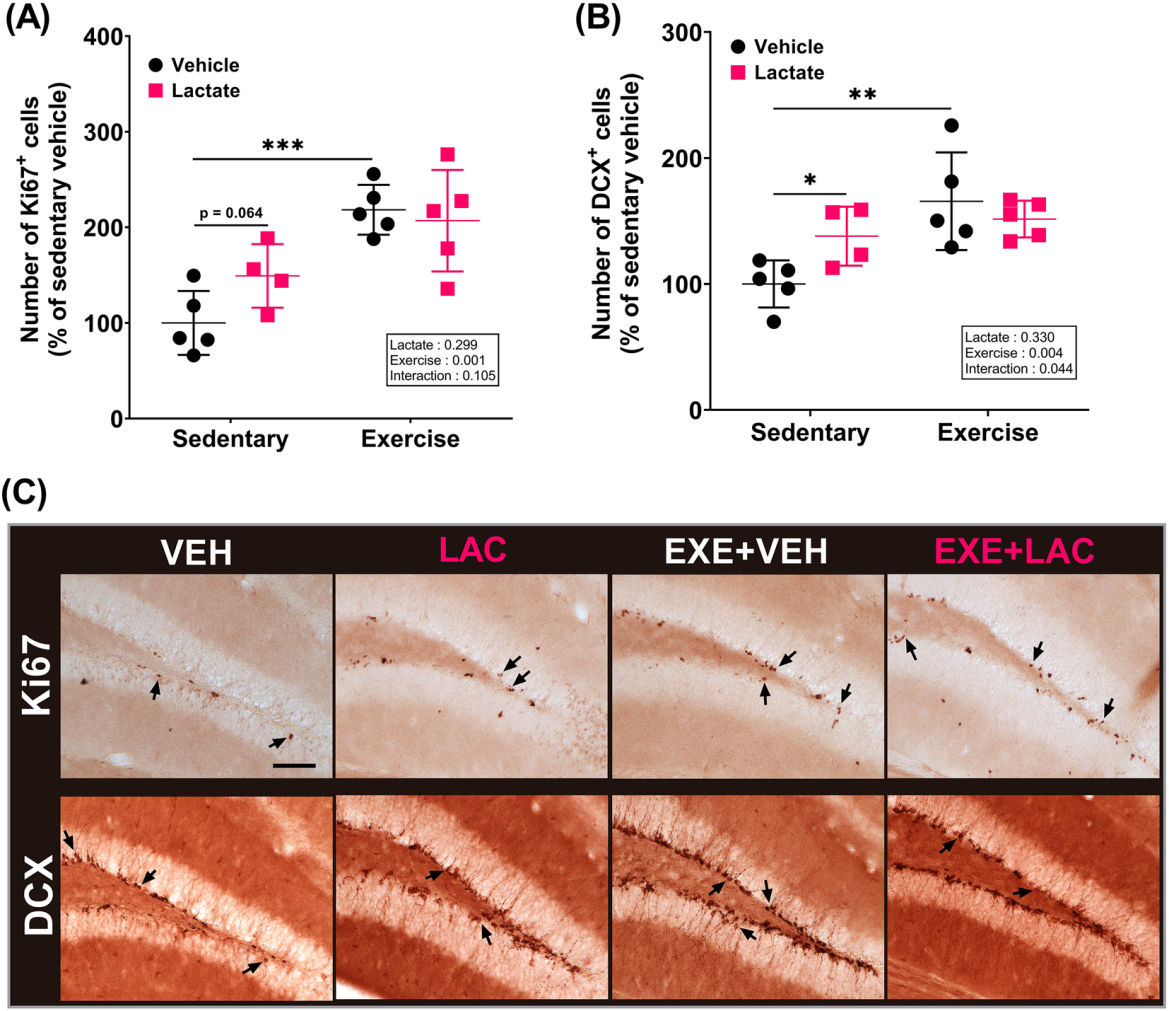
Exercise with lactate administration did not augment exercise-induced adult hippocampal neurogenesis, but lactate administration promoted adult hippocampal neurogenesis. (**A**) Quantification of Ki67-positive cell and (**B**) of DCX-positive cell in subgranular zone of dentate gyrus of mice. (**C**) The represent image of immunohistochemistry. Arrow indicates a represent positive cell. Scale bar: 100 μm. Two-way ANOVA was performed, and independent Student’s *t*-test was used for post hoc test (*n* = 4–5 per group). *VEH* sedentary without lactate, *LAC* sedentary with lactate, *EXE + VEH* exercise without lactate, *EXE + LAC* exercise with lactate; *p < 0.05, **p < 0.01, and ***p ≤ 0.005. Data are presented as the mean ± standard deviation.

To validate the effect of exercise with lactate intake on AHN, we evaluated proliferation and neuronal differentiation of the neurogenic pool in the DG. After 5 weeks of exercise training and/or lactate administration five times per week in 7-week-old male ICR mice (Fig. 2A), the number of Ki67^+^ (cell proliferation marker) and doublecortin (DCX, immature neuronal marker)^+^ cells in the exercise without lactate group (EXE + VEH) was significantly higher than that in the sedentary without lactate group (VEH) (Fig. 3A–C; *p* = 0.001 and *p* = 0.009, respectively). The number of Ki67^+^ cells in the sedentary with lactate intake group (LAC) indicated a slight increase compared to that in the VEH group (Fig. 3A; *p* = 0.064), and the number of DCX^+^ cells in the LAC significantly increased compared to VEH (Fig. 3B; *p* = 0.03). However, there was no difference between EXE + VEH and the exercise with lactate intake group (EXE + LAC) in the number of Ki67^+^ and DCX^+^ cells (Fig. 3A–C). Therefore, these results indicate that exercise with lactate intake did not augment exercise-induced AHN, although both exercise and lactate promoted AHN independently.

### Exercise with lactate administration augmented exercise-induced improvement in reference and retention memory

**Figure 4:**
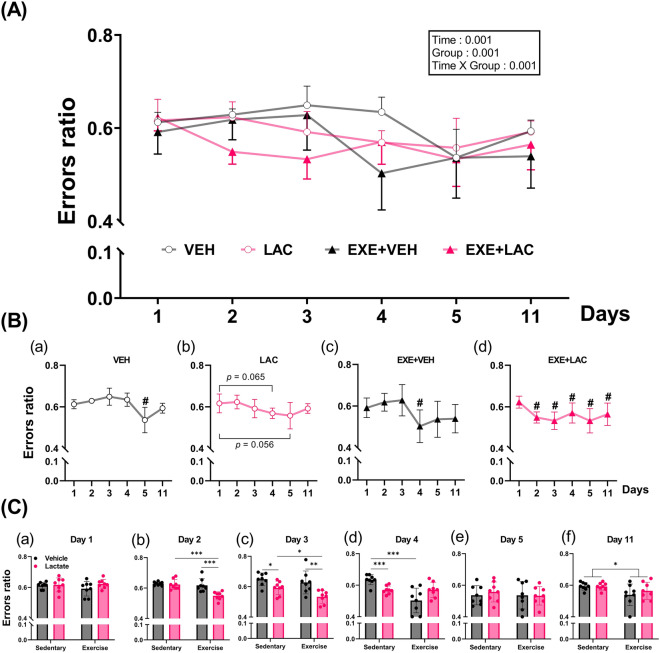
Exercise with lactate administration augmented exercise-induced improvement in reference and retention memory. The test was conducted using eight-arm radial arm maze (RAM). Day 1 to 5 is learning phase of RAM task and Day 11 is retention memory trial of RAM task. (**A**) The entire results of RAM task. Two-way repeated ANOVA was performed. In order to improve readability of indications of post hoc results, (**B**) the results of comparison of changes over time within the same group (paired *t*-test was used for post hoc test) and (**C**) the results of comparison of difference among groups within the same day (independent Student’s *t*-test was used for post hoc test) are separately presented. *VEH* sedentary without lactate, *LAC* sedentary with lactate, *EXE + VEH* exercise without lactate, *EXE + LAC* exercise with lactate; *n* = 8 per group, *p < 0.05, **p < 0.01, and ***p ≤ 0.005; ^#^*p* < 0.05, vs day 1 within group. Data are presented as the mean ± standard deviation.

To examine the effect of exercise with lactate intake on reference and retention memory, another cohort of mice was used (Fig. 2B) and performed an eight-arm radial arm maze (RAM). Of note, the entire results are presented in Fig. 4A, and for readability of indications of post hoc test we subdivide the results and presented in Fig. 4Ba–d (comparison of changes over time within the same group) and in Fig. 4Ca–f (comparison of difference among groups within the same day).

In the learning phase (day 1–5) of the task, the appearance of learning curves was different among groups. On day 1, the performance of RAM task did not differ among groups [Fig. 4Ca; two-way ANOVA: lactate (*p* = 0.192), exercise (*p* = 0.590) and interaction (*p* = 0.319)]. On day 2, however, errors ratio was reduced only in EXE + LAC compared to day 1 (Fig. 4Bd; *p* = 0.001). EXE + LAC showed significant improvement in reference memory compared to other groups also (Fig. 4Cb; EXE + VEH vs. EXE + LAC: *p* = 0.002; LAC vs. EXE + LAC: *p* = 0.001). In turn, the group showed the second-best performance in reference memory was EXE + VEH (Fig. 4Bc; day 1 vs. day 4: *p* = 0.023) and LAC (Fig. 4Bb; day 1 vs. day 4: *p* = 0.065, day 1 vs. day 5: *p* = 0.056) (Fig. 4Cd; VEH vs. LAC: *p* = 0.001, VEH vs. EXE + VEH: *p* = 0.001), and the next was VEH (Fig. 4Ba; day 1 vs. day 5: *p* = 0.022). Finally, on day 5, there was no difference in reference memory among groups [Fig. 4Ce; two-way ANOVA: lactate (*p* = 0.712), exercise (*p* = 0.612) and interaction (*p* = 0.619)].

In the retention memory trial of the task (day 11), the improved reference memory was significantly retained only in EXE + LAC (Fig. 4Bd; day 1 vs. day 11: *p* = 0.049). However, there was no difference in retention memory among groups although significant main effect of exercise was confirmed [Fig. 4Cf; two-way ANOVA: lactate (*p* = 0.483), exercise (*p* = 0.02) and interaction (*p* = 0.426)].

Collectively, these results indicate that exercise with lactate administration augmented exercise-induced improvement in reference and retention memory.

### Exercise with lactate administration promoted spatial working memory

**Figure 5:**
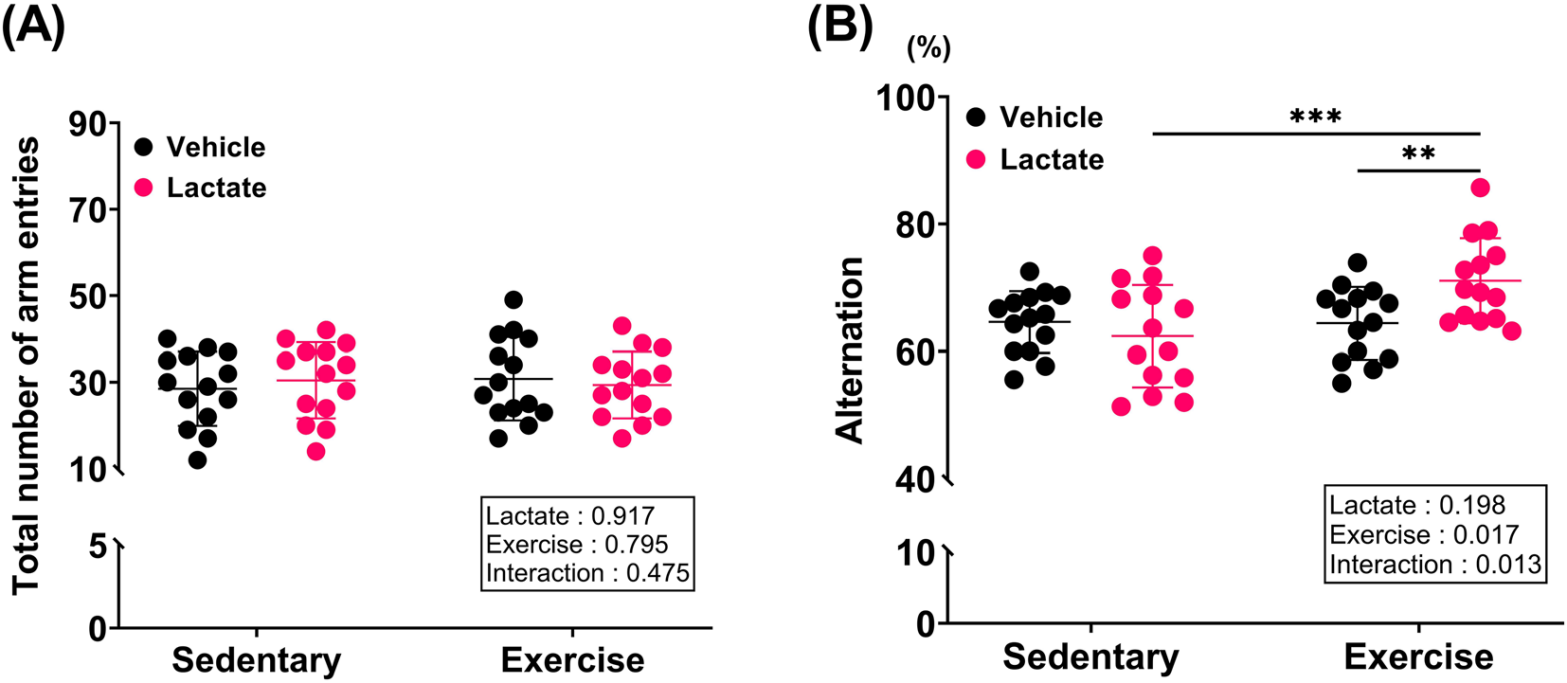
Exercise with lactate administration promoted spatial working memory. The test was conducted using Y-maze. (**A**) The total number of arm entries and (**B**) spontaneous alternation behavior (spatial working memory). Two-way ANOVA was performed, and independent Student’s *t*-test was used for post hoc test (*n* = 14 per group). **p < 0.01 and ***p ≤ 0.005. Data are presented as the mean ± standard deviation.

Also, we performed Y-maze test to measure spatial working memory. The ability to alternate between two arms of the maze requires mice to know which arms have already been visited. Therefore, alternation behavior can be regarded as a measure of spatial working memory, which is a hippocampus-related cognitive function. The total number of arm entries did not differ among the groups (Fig. 5A), which indicated that there was likely no bias in the alternation that could exist when the total number of arms entered was unequal. Nevertheless, alternation in EXE + LAC was significantly higher than that in EXE + VEH and LAC (*p* = 0.004 and *p* = 0.009, respectively; Fig. 5B), and there was no difference between VEH and either LAC or EXE + VEH (Fig. 5B). As a result, exercise with lactate administration promoted spatial working memory.

### Exercise with lactate administration effectively enhanced hippocampal FNDC5, BDNF, PGC1α, and MCT2 protein expression

To understand the molecular changes resulting from exercise with lactate intake, we investigated the expression of the following proteins relevant to AHN in the context of exercise and lactate effects: FNDC5, BDNF, peroxisome proliferator-activated receptor gamma, coactivator 1 alpha (PGC1α), MCT2, and MCT1.

**Figure 6:**
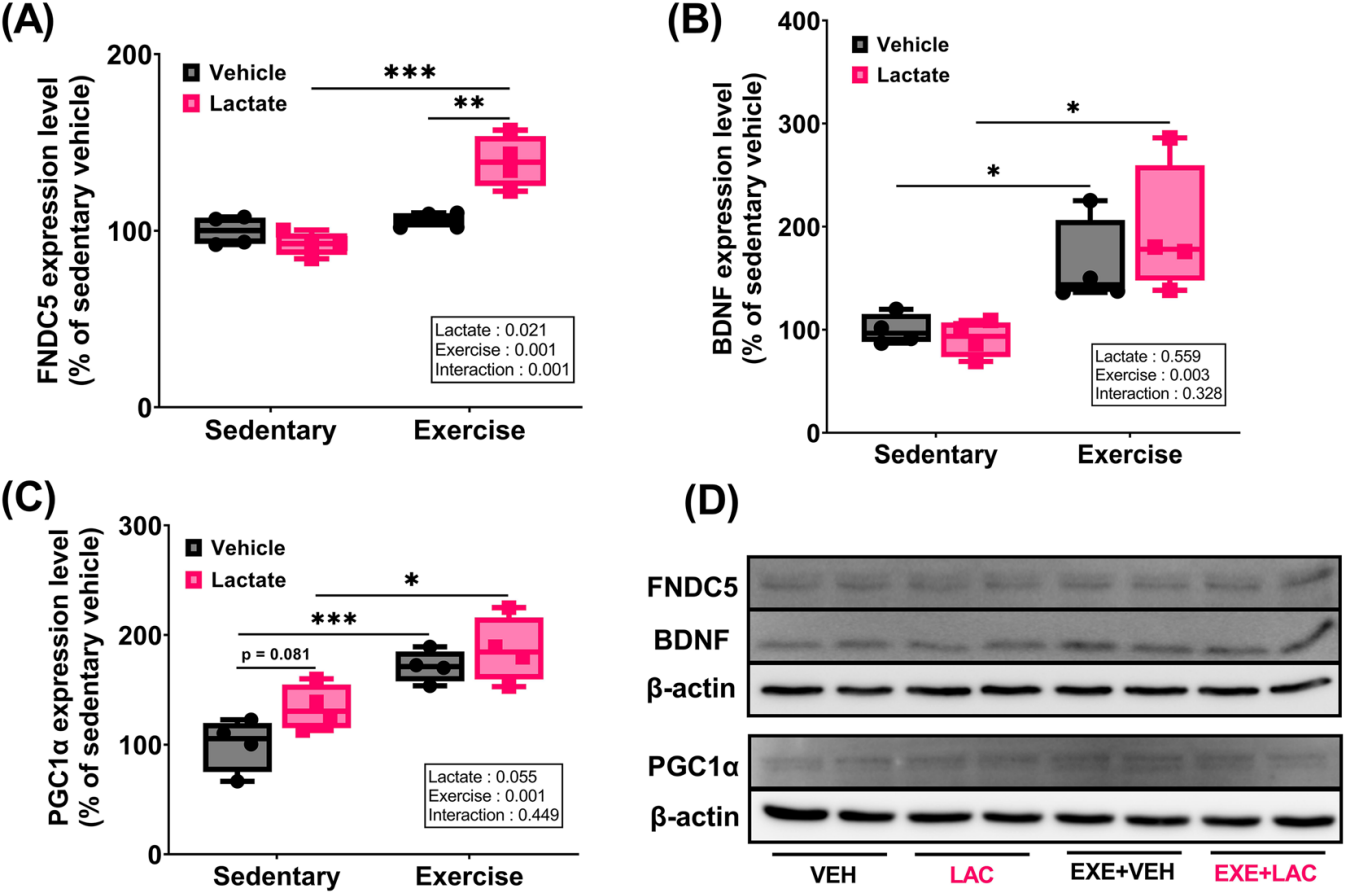
Exercise with lactate administration effectively enhanced the hippocampal molecules relevant to exercise-induced adult hippocampal neurogenesis. Level of protein expression of hippocampal (**A**) FNDC5, (**B**) BDNF, and (**C**) PGC1α in mice. (**D**) The represent image of western blot. Two-way ANOVA was performed, and independent Student’s *t*-test was used for post hoc test (*n* = 4 per group). The original blots are presented in Supplementary Figs. 1 and 2. *VEH* sedentary without lactate, *LAC* sedentary with lactate, *EXE + VEH* exercise without lactate, *EXE + LAC* exercise with lactate, *FNDC5* fibronectin type III domain-containing protein 5, *BDNF* brain derived neurotrophic factor, *PGC1α* peroxisome proliferator-activated receptor gamma coactivator 1-alpha; *p < 0.05, **p < 0.01, and ***p ≤ 0.005. Data are presented as box plot.

**Figure 7:**
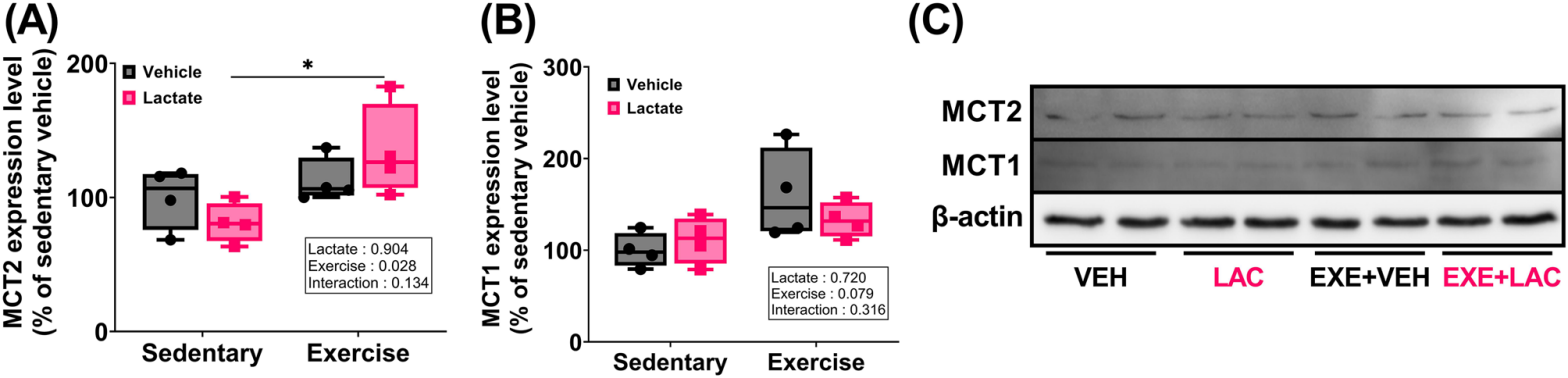
Exercise with lactate administration effectively enhanced the hippocampal MCT2 protein expression but not MCT1. Level of protein expression of hippocampal (**A**) MCT2 and (**B**) MCT1 in mice. (**C**) The represent image of western blot. Two-way ANOVA was performed, and independent Student’s *t*-test was used for post hoc test (*n* = 4 per group). The original blots are presented in Supplementary Fig. 3. *VEH* sedentary without lactate, *LAC* sedentary with lactate, *EXE + VEH* exercise without lactate, *EXE + LAC* exercise with lactate, *MCT1/2* monocarboxylate transporter 1/2; *p < 0.05. Data are presented as box plot.

Exercise significantly affected the expression of all relevant proteins [Fig. 6A–D; two-way ANOVA; FNDC5 (*p* = 0.001), BDNF (*p* = 0.003), PGC1α (*p* = 0.001); Fig. 7A; MCT2 (*p* = 0.028)], except MCT1 (Fig. 7B,C; two-way ANOVA; *p* = 0.079). Lactate intake did not affect the hippocampal FNDC5, BDNF (Fig. 6A,B), and MCT2 (Fig. 7A) protein expression. However, the hippocampal PGC1α protein expression in LAC tended to be higher than that in VEH (Fig. 6C; *p* = 0.08). Notably, the hippocampal FNDC5 protein expression in EXE + LAC was significantly elevated compared to EXE + VEH (Fig. 6A; *p* = 0.007), and the hippocampal BDNF, PGC1α, and MCT2 protein expression in EXE + LAC was higher than that in EXE + VEH by 20%, 9%, and 19%, respectively, although the difference was not statistically significant (Figs. 6B,C and 7A).

### Exercise, lactate, and co-treatment did not affect hippocampal VEGFA or HCAR1 protein expression

**Figure 8:**
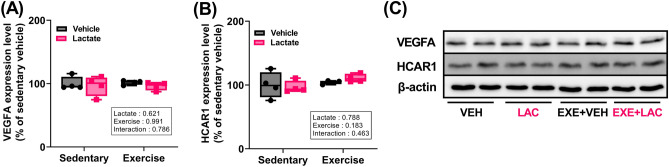
Exercise, lactate, and co-treatment did not affect hippocampal VEGFA and HCAR1 protein expression. Level of protein expression of hippocampal (**A**) VEGFA and (**B**) HCAR1. (**C**) The represent image of western blot. Two-way ANOVA was performed, and independent Student’s *t*-test was used for post hoc test (*n* = 4 per group). The original blots are presented in Supplementary Figs. 4 and 5. *VEH* sedentary without lactate, *LAC* sedentary with lactate, *EXE + VEH* exercise without lactate, *EXE + LAC* exercise with lactate, *VEFGA* vascular endothelial growth factor A, *HCAR1* hydroxycarboxylic acid receptor 1. Data are presented as box plot.

To further elucidate the proteins relevant in the context of exercise- and lactate-mediated effects on AHN and cognitive behavior in EXE + LAC, we investigated the angiogenesis-related proteins vascular endothelial growth factor A (VEGFA) and hydroxycarboxylic acid receptor 1 (HCAR1). We did not find any difference in hippocampal VEGFA and HCAR1 protein expression among the groups (Fig. 8A–C).

## Discussion

Lactate has been implicated as a major molecule in mediating exercise-induced AHN. Accordingly, we reasoned that exogenous lactate intake could partially mimic the effect of exercise on AHN. Therefore, we hypothesized that exercise with oral intake of lactate augments exercise-induced AHN. To validate this assumption, we examined the effect of exercise with lactate intake on the proliferation and neuronal differentiation of the neurogenic pool in the DG, reference and retention memory, spatial working memory, and the expression of relevant hippocampal proteins in mice.

MCTs presents in endothelial cells of blood–brain barrier^28^, which indicates that circulating lactate can transport into the central nervous system. Indeed, circulating lactate transports into the central nervous system and is utilized in the brain both in human^26^ and rodents^27^. Furthermore, MCTs are particularly abundant in the hippocampus compared to other regions of brain^28^, which indicates that the hippocampus is susceptible to the circulating lactate. Indeed, in the previous study measuring the hippocampus extracellular lactate concentration by microdialysis after intraperitoneal injection of lactate, time course changes in the hippocampal extracellular lactate concentration occurred simultaneously with the time course changes in blood lactate concentration, and the elevation in the hippocampal extracellular lactate level is blocked by MCT inhibitor^25^. Elevation in blood lactate concentration to more than 6 mM was sufficient to result in a significant increase in the hippocampal lactate levels^22,25^. Therefore, we assert that oral administration of 3 g/kg lactate in our study is enough to increase the hippocampal lactate concentration; thus, our applied treatment can affect the hippocampus.

A previous study showed that intraperitoneal injection of lactate promotes AHN in an MCT2-dependent manner^23^. Similarly, in the present study, we observed that lactate enhances proliferation and neuronal differentiation of the neurogenic pool in the DG (Fig. 3). However, lactate did not augment exercise-induced AHN (Fig. 3).

The lack of an augmented effect of exercise with lactate on AHN may be related to the physiologically limited capacity of neurogenesis, i.e., an upper limit to the increase in neurogenesis. In several typical rodent strains, an approximate 1.5- to 2.0-fold increase in neurogenesis by exercise compared to baseline seems to be the upper limit^8,32,36,37^. In previous studies, the exercise protocol was usually set at 10–15 m/min (velocity) for 40–60 min (duration), 5 days/week (frequency) over a period of 4 weeks. Considering these observations, the exercise protocol we used (15–25 m/min for 40–50 min, 5 days/week for 5 weeks) should induce a sufficient increase in AHN because the amount of exercise in our study is higher compared to the previous studies. Indeed, in the current study exercise increased the number of Ki67- and DCX-positive cells in the DG by 2- and 1.5-fold, respectively, compared to the control condition (Fig. 3). Thus, there may be little (or no) opportunity for neurogenesis induced by lactate, as exercise had a substantial effect on neurogenesis. This interpretation is partially supported by previous studies using mice with a lower than normal level of neurogenesis, where mice with abnormal conditions of neurogenesis showed more potential for increased neurogenesis compared to mice in normal conditions. Indeed, in previous studies, AHN impeded by a chronic stressful environment^6^ and aging^38^ was significantly increased to a greater extent by co-treatment of exercise and a supplement than by exercise alone; additionally, it is noteworthy that the exercise intensity used was lower than that used in the current study. Therefore, we suggest that further investigation focusing on level of exercise intensity and/or pathological models is required to validate the augmented effect of exercise with lactate on AHN.

While we were not able to observe an augmented effect of exercise with lactate on AHN, we found that exercise-induced improvement in reference and retention memory were augmented by lactate (Fig. 4). And spatial working memory was promoted by co-treatment (Fig. 5), also hippocampal FNDC5, BDNF, PGC1α (Fig. 6A), and MCT2 (Fig. 7A) protein expressions were effectively enhanced by co-treatment. We speculate that this additive effect of co-treatment on memory and the relevant factors may be explained by neuronal plasticity rather than neurogenesis alone.

Neuronal plasticity occurs at the cellular level during learning and memory^18^. A previous study identifying the action of lactate on neuronal plasticity showed that lactate promoted the expression of synaptic plasticity-related genes (*Arc*, *c-Fos*, and *Zif268*) both in primary neuronal cultures of the mouse neocortex and in vivo^39^. This result was corroborated by transcriptome analysis identified that expression of 15 neuronal plasticity-related genes was upregulated by lactate in primary cultures of cortical neurons^40^. Furthermore, the beneficial effect of lactate on neuronal plasticity^39^ and memory^15^ was negated by MCT inhibition. This finding suggests the promoting effect of lactate on neuronal plasticity and the importance of MCTs when lactate acts as a promoter of neuronal plasticity.

Brain FNDC5 plays a significant role in neuronal plasticity, especially in the context of exercise^41,42^. Hippocampal neuronal function was impaired by the knockdown of brain *Fndc5* in wild-type mice, and impaired hippocampal neuronal plasticity in an AD model was rescued by boosting brain FNDC5. Finally, the study showed that exercise-induced improvement in hippocampal neuronal function was blunted by the downregulation of brain FNDC5 expression; these results indicate the role of brain FNDC5 as a key mediator of exercise on neuronal plasticity and memory^41^.

Hippocampal BDNF is an important molecule of effects of exercise on many aspects of both AHN^19^ and neuronal plasticity^18^. Hippocampal BDNF is regulated via several pathways; however, in the context of exercise, it has been reported that a PGC1α/FNDC5-dependent mechanism is an important way to regulate hippocampal BDNF^42^. This previous study showed that the knockdown of PGC1α significantly downregulated hippocampal *Fndc5* gene expression both in vitro and in vivo. Hippocampal *Bdnf* gene expression was significantly upregulated by forced expression of FNDC5 both in vitro and in vivo. Notably, the expression of important neuronal plasticity-related genes (*Arc*, *c-Fos*, *Npas4*, *Zif268*) was also sharply increased by forced expression of FNDC5 both in primary cultures of hippocampal neurons and in the hippocampus of wild-type mice. These results demonstrate that hippocampal BDNF affects hippocampal neuronal plasticity in a PGC1α/FNDC5-dependent manner.

Consequently, the improvement in memory by exercise with lactate intake (Figs. 4 and 5) may have resulted from enhanced neuronal plasticity due to augmented hippocampal FNDC5 protein expression and small increase in hippocampal BDNF, PGC1α, and MCT2 protein expression (Figs. 6 and 7A). Additionally, HCAR1 is a lactate receptor abundant around cerebral blood vessels (but sparsely expressed in skeletal muscles) and is involved in stimulating the exercise-induced cerebral VEGFA expression and angiogenesis^43^. It has been suggested that the beneficial effect of exercise on brain functions partially results from improved cerebral perfusion via angiogenesis^44^. Thus, our speculation that the improvement in memory by exercise with lactate intake may results from the enhanced neuronal plasticity is partially supported by the finding that there was no change in hippocampal VEGFA and HCAR1 protein expression either by exercise or lactate (Fig. 8).

In summary, our study shows that exercise with lactate did not augment exercise-induced AHN. However, the physiologically limited capacity for neurogenesis in normal mice coupled with the robust neurogenesis response to exercise may have occluded the potential contribution of lactate. Nevertheless, exercise with lactate augmented exercise-induced improvement in reference and retention memory. Also, spatial working memory was promoted by co-treatment. Consistent with this result, hippocampal FNDC5 protein expression was significantly augmented, and hippocampal BDNF, PGC1α, and MCT2 protein expression were slightly more upregulated by exercise with lactate compared to exercise (the changes were not statistically significant). These positive changes are likely to result in enhancing hippocampal neuronal plasticity and, subsequently, may induce the improvement in memory.

Herein, we partially evaluated AHN (proliferation and neuronal differentiation) and did not explore neuronal maturation, i.e., the final phase of neurogenesis. Investigating neuronal maturation requires tracing methods such as injection of bromodeoxyuridine. Considering that AHN is vulnerable to stress, we decided not to use this method to avoid confounds from excessive stress that can occur when both oral and intraperitoneal injections are used. Therefore, an experiment that specifically evaluates neuronal maturation phase is needed to establish a better understanding of the complete effect of lactate on AHN.

Lactate is a highly relevant signaling molecule that regulates brain functions^14^. However, it is unknown whether lactate is a primary mediator of exercise-induced AHN. Furthermore, it is unknown whether lactate acts equally even under exercise conditions that differ physiologically from resting conditions in the brain. So far there is no critical evidence that exercise-induced AHN is mediated by lactate. This aspect may be a major limitation of the present study. Therefore, we plan to conduct further studies to find direct evidence to reveal the relationship between lactate and exercise-induced AHN.

In conclusion, to our knowledge, this is the first study to investigate the effect of co-treatment of exercise and lactate on AHN. We demonstrated the effect of lactate on AHN via oral administration, i.e., through an applied approach rather than an invasive approach. Our results suggest that lactate has a potential to be developed as a novel supplement that improves the positive effects of exercise on the hippocampus and its cognitive function.

## Methods

### Ethical approval

The animal study was reviewed and approved by the Konkuk University Institutional Animal Care and Use Committee (No. KU19149). All methods were performed in accordance with the relevant guidelines and regulations. The study was carried out in compliance with the ARRIVE guidelines: https://arriveguidelines.org/. Furthermore, efforts were made to minimize discomfort and stressful situations.

### Animals

Before starting the experiment, 6-week-old male ICR mice (33.2 ± 1.4 g, Orient Bio Inc., Seongnam, Republic of Korea) were habituated to the laboratory environment for at least a week. All mice were housed in standard transparent plastic cages under a controlled temperature at 23–25 °C with 40%–50% humidity and a 12-h light/dark cycle (lights on: 07:00–19:00). Standard chow diet (Orient Bio Inc., Seongnam, Republic of Korea) and water were provided ad libitum.

### Pilot experiment

In the pilot experiment with 6-week-old male ICR mice distinct from a set of mice used in the main experiment (cohort 1 and cohort 2), blood lactate concentration was measured using a lactate analyzer (LT-1730, Lactate Pro 2, ARKRAY, Kyoto, Japan) after a single oral administration of lactate over time or immediately after exercise from tail vein blood at D1, D15, and D29 (Fig. 1).

### Experimental design

This study comprised two independent experiments except the pilot test. Mice of experiment 1 were mainly for biochemical analysis (Fig. 2A, n = 9 per group) and mice of experiment 2 were for behavioral analysis (Fig. 2B, n = 8 per group). Mice were randomly divided into four groups: VEH, LAC, EXE + VEH and EXE + LAC. LAC mice were orally administered 3 g/kg of sodium lactate, which was a mixture of a stock solution of sodium lactate (195–05,965, Wako Chemical, Osaka, Japan) and distilled water at a ratio of 1:1, and VEH mice were administered an equal solution excluding sodium lactate. EXE mice were administered the solution immediately after every exercise training.

Exercise training was conducted five times per week for five weeks. The treadmill exercise was performed at 15 m/min for 40 min in the first week, 20 m/min for 40 min in the second week, 22 m/min for 50 min in the third week, and 25 m/min for 50 min in the fourth and fifth weeks. The treadmill slope was fixed at 8° (Fig. 2). For motivating mice to run, mild electrical stimulation on a grid at the rear of the treadmill was given. Electrical stimulation was set at a constant current of 0.4 mA, which is appropriate electrical level not to cause major distress^45–47^ and not to increase the circulating lactate level (Fig. 1B).

### Tissue processing

Mice were dissected under deep anesthesia with 10 μL/g of 1.25% avertin, 48 h after the last treadmill exercise and lactate administration. The reason that mice are sacrificed 48 h after the treatments is to avoid acute effects of exercise and/or lactate on hippocampal protein and/or mRNA expression. Considering the previous studies, single exercise and/or lactate injection can increase hippocampal protein and/or mRNA expression in 12 h including BDNF, PGC1α, and so on^22,25^. Mice were transcardially perfused with cold 0.9% saline. For immunohistochemistry, we randomly selected 5 out of 9 mice of experiment 1 and brains were removed, postfixed in 4% paraformaldehyde in 0.1 M phosphate-buffered saline (PBS) for 48 h, and stored in cold 30% sucrose in 0.1 M PBS until completely sunken. Brains were then cryosectioned into coronal 40-µm-thick slices (at this stage, one brain sample of LAC was damaged, thus we excluded it from the results); the slices were stored in a cryoprotectant solution (30% ethylene glycol + 30% glycerol in 0.1 M phosphate buffer) at − 20 °C until further analysis. For immunoblotting, bilateral hippocampi of the other 4 mice of experiment 1 were dissected on an ice-cooled plate, immediately frozen in liquid nitrogen, and stored at − 80 °C until further analysis.

### Immunohistochemistry

Every fourth section was taken from the region between brain bregma − 1.46 mm and − 2.18 mm. Six randomly selected sections per brain were used for analysis. Free-floating sections were incubated in 0.3% H_2_O_2_ to inhibit endogenous peroxidase and in 10% normal goat serum (NGS, S-1000, Vector Laboratories, Burlingame, CA, USA) prepared in PBS containing 0.1% Tween 20 (PBS-T) to block nonspecific protein binding. Sections were incubated overnight at 4 °C with rabbit anti-Ki-67 (1:1,000, ab15580) and rabbit anti-DCX (1:2000, ab18723) primary antibodies (Abcam, Cambridge, MA, USA) in 3% NGS prepared in 0.1% PBS-T and subsequently for 1 h at 23–25 °C in biotinylated goat anti-rabbit secondary antibodies (1:300, BA-1000, Vector Laboratories, Burlingame, CA, USA) in 3% NGS prepared in 0.1% PBS-T. The sections were further incubated with ABC reagent (1:200, VECTASTAIN Elite ABC kit, PK-6101; Vector Laboratories, Burlingame, CA, USA) for 90 min at 23–25 °C. Finally, the sections were visualized using a DAB Substrate Kit (SK-4100, Vector Laboratories, Burlingame, CA, USA) and mounted. To determine the subgranular zone of the DG, the granule cell layer was divided into three layers^48^. The granule cell layer width was determined using the gridlines. Then, the granule cell layer was divided into three layers of approximately equal thickness. The number of positive cells in the most inner layer was manually counted using EVOS M5000 microscopy (Thermo Fisher Scientific, Waltham, MA, USA) under 20 × and 40 × objective lenses and normalized by length of the border line between the subgranular zone and hilus. The length was measured using Image J software (NIH Image Engineering, Bethesda, MD, USA).

### Immunoblotting analysis

Hippocampi were homogenized using a TissueRuptor (QIAGEN, Hilden, Germany) in 400 μL of protein extraction buffer (EzRIPA Lysis kit, WSE07420, ATTO, Tokyo, Japan). Lysates were centrifuged at 20,000×*g* at 4 °C for 15 min. Thereafter, the lipid layer (top layer) was removed, and clear supernatants were transferred to a new tube. The supernatants were centrifuged again at 20,000×*g* at 4 °C for 15 min. Finally, the supernatants were transferred to new tubes. Protein concentration was determined using a Pierce™ BCA Protein Assay Kit (23225, Thermo Fisher Scientific, Waltham, MA, USA). Proteins were denatured by heating at 100 °C for 5 min. Total protein (40 μg per lane) was separated using 10% or 12% SDS-PAGE at 60 V for 30 min, followed by 100 V for 120 min, and then transferred to polyvinylidene difluoride membranes (ISEQ00010, Millipore, Billerica, MA, USA) at 100 V for 2 h. The membranes were blocked for 1 h at 23–25 °C in 5% non-fat dried milk (NFDM, F141511, Cellconic, Hanam, Republic of Korea) in 0.1% PBS-T, then incubated overnight at 4 °C in primary antibodies in 3% NFDM in 0.1% PBS-T, and subsequently incubated for 90 min at 23–25 °C in horseradish peroxidase-conjugated secondary antibodies in 3% NFDM in 0.1% PBS-T (the information on antibodies is provided as Supplementary Table 1). Immunodetection was performed using ECL™ Prime western blotting Detection Reagents (GERPN2232; Cytiva, Marlborough, MA, USA). All images showing the results of the quantitative analysis were assessed using the ImageJ software.

### Radial arm maze

To measure reference and retention memory, eight-arm radial arm maze (RAM)^49,50^ was performed with mice of cohort 2 (Fig. 2B). To acclimatize to the maze and a reward (sunflower seeds)^7,50^, mice were allowed to explore and feed freely in the RAM 30 min once a day for 3 days. The rewards were scattered in all arms. The learning phase was started following the acclimatizing phase. During the learning phase, each mouse was performed one trial daily for 5 days. The same three arms were rewarded each day and across trials. The arms placed to be rewarded were never changed for a given mouse but varied among mice. A trial ended when 5 min had elapsed or all the rewards had been received, whichever occurred first. The light was kept dim during all trial to reduce the anxiety of mice. Entry into a never-rewarded arm was considered a reference memory error. Therefore, errors ratio refers to the entry number of reference memory errors divided by the total entry number. Retention memory test was conducted 6 days after the last learning trial.

### Spontaneous alternation behavior test using Y-maze

To measure spatial working memory, the spontaneous alternation behavior test was conducted with all mice of experiment 1 and 2 (Fig. 2). Each mouse was randomly placed in one arm of the symmetrical Y-maze and allowed to explore freely for 6 min. The light was kept dim during test to reduce the anxiety of mice. The sequence and total number of arms entered were recorded except for the first 1 min, which was considered as the habituation period. The number of arm entries was counted when the hind paws of the mouse were completely placed in the arm. An alternation was defined only as entries into all three arms on consecutive occasions. Therefore, the number of maximum alternations was the total number of arm entries minus two, and the percentage of alternations was calculated as (actual alternations/maximum alternations) × 100. Additionally, in case of mice that recorded 3 or less the total number of arm entries, we were not able to obtain data. Finally, 14 out of 17 mice were included in the results.

### Statistical analysis

All data were analyzed using IBM SPSS Statistics 25 software. Graph construction was performed using the GraphPad Prism software (version 9.0). All data were checked for normality of distribution using the Shapiro–Wilk test, and all data were verified for normality, except for blood lactate concentration data of LAC at 120 min (Fig. 1A). Therefore, a comparison of LAC and VEH at 120 min was performed using a two-tailed Mann–Whitney test. For other data with normal distribution, comparison of two or more groups over time was performed using two-way repeated analysis of variance (ANOVA), and post hoc tests were performed using one-way repeated ANOVA, paired *t*-test, or independent Student’s *t*-test. Comparisons between two groups were performed using an independent Student’s *t*-test. Comparisons of four groups were performed using two-way ANOVA, and post hoc tests were performed using an independent Student’s *t*-test. Blood lactate concentration (Fig. 1), immunohistochemistry (Fig. 3), RAM (Fig. 4) and Y-maze (Fig. 5) data are presented as the mean ± standard deviation (SD), and immunoblotting data are presented as box plots (Fig. 6-8). A value of *p* < 0.05 was considered statistically significant.

## Supplementary Information

Supplementary Information.

## Data Availability

The datasets generated and/or analyzed during the current study are available from the corresponding author upon reasonable request.
