# Supplementary material for: Exogenous lactate augments exercise-induced improvement in memory but not in hippocampal neurogenesis
Source: Sci Rep. 2023 Apr 10;13:5838. doi: 10.1038/s41598-023-33017-1 (PMC10086059; doi:10.1038/s41598-023-33017-1)
Supplement: Supplementary file 1 — Supplementary Information. [file 41598_2023_33017_MOESM1_ESM.pdf]

Exogenous Lactate Augments Exercise-Induced Improvement in Memory but not in Hippocampal Neurogenesis

Deunsol Hwang[1,2,†], Jisu Kim[1,2,†], Sunghwan Kyun[1,2], Inkwon Jang[1,2], Taeho Kim[1,2], Hun-Young Park[1,2], and Kiwon Lim[1,2,3,*]

[1]Laboratory of Exercise and Nutrition, Department of Sports Medicine and Science in Graduate School, Konkuk University, Seoul, the Republic of Korea

[2]Physical Activity and Performance Institute (PAPI), Konkuk University, Seoul, the Republic of Korea

[3]Department of Physical Education, Konkuk University, Seoul, the Republic of Korea

[*]exercise@konkuk.ac.kr

[†]These authors have contributed equally to this work

Supplementary Table 1. Information of used antibodies for immunoblotting in the experiment.

Primary antibody				Secondary antibody		
Target	Molecular Weight (kDa)	Concentration	Cat.No (manufacturer)	Target	Concentration	Cat.No (manufacturer)
FNDC5	24, 48	1:1000	ab174833 (Abcam)	anti-rabbit	1:2000	sc-2357 (Santa Cruz Biotechnology)
BDNF	15	1:1000	ab108319 (Abcam)		1:4000	
PGC1α	92 - 105	1:1000	ab54481 (Abcam)		1:2000	
MCT2	43	1:200	sc-166925 (Santa Cruz Biotechnology)	anti-mouse	1:1000	sc-516102 (Santa Cruz Biotechnology)
MCT1	54	1:500	ab93048 (Abcam)	anti-rabbit	1:1000	sc-2357 (Santa Cruz Biotechnology)
VEGFA	23, 45	1:1000	ab46154 (Abcam)		1:10000	
HCAR1	40	1:2000	NLS2095 (NONUS Biologicals)		1:4000	
beta-actin	43	1:2000	sc-47778 (Santa Cruz Biotechnology)	anti-mouse	1:4000	sc-516102 (Santa Cruz Biotechnology)

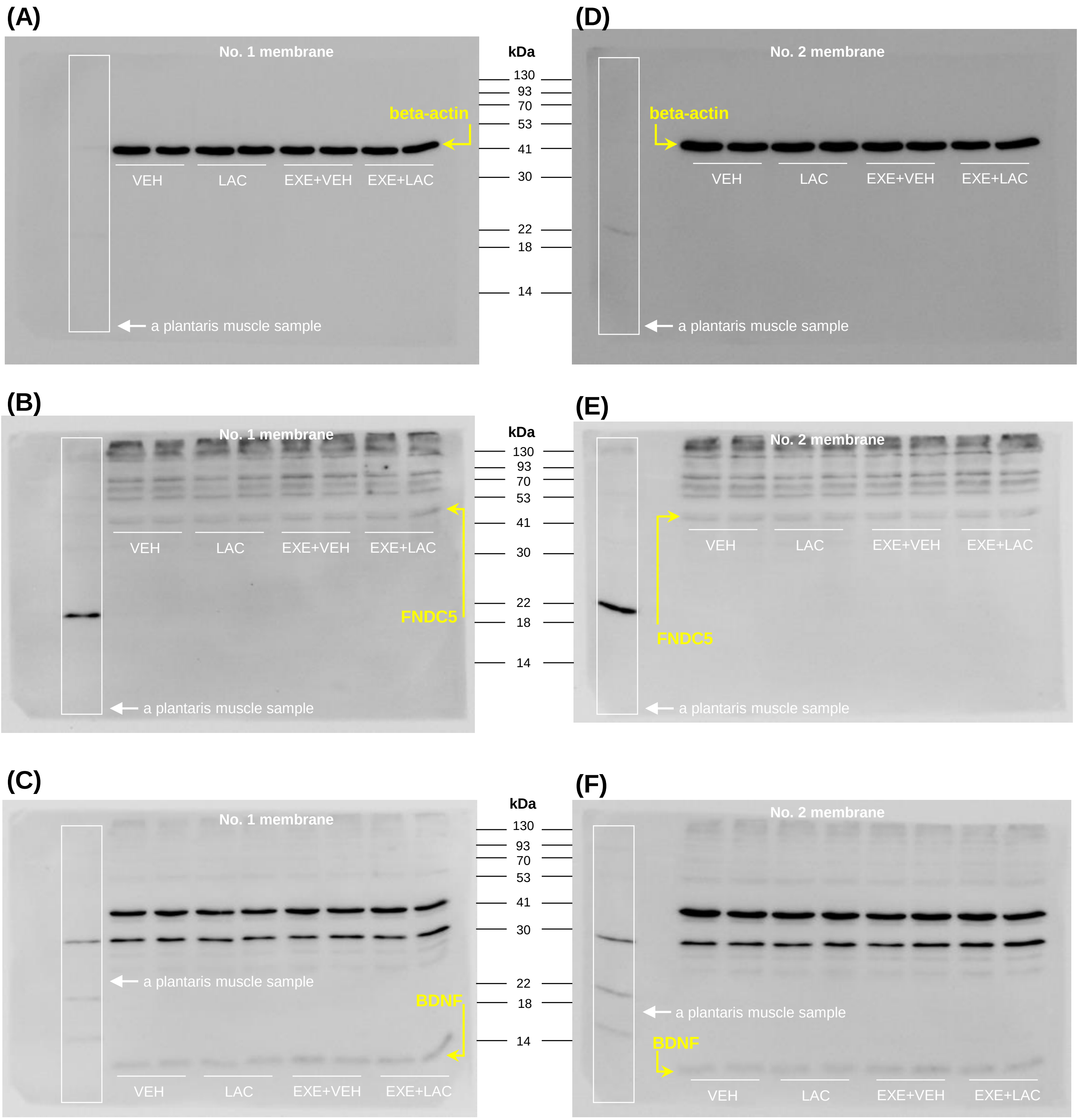

Supplementary Figure 1. The original blots of hippocampal protein expression of FNDC5 and BDNF that presented in Fig. 5D. The result of **(A)** beta-actin, **(B)** FNDC5, and **(C)** BDNF is obtained from No. 1 membrane. The result of **(D)** beta-actin, **(E)** FNDC5, and **(F)** BDNF is obtained from No. 2 membrane. To check analysis conditions of plantaris muscle for another study, one plantaris muscle sample was running together. VEH, sedentary without lactate; LAC, sedentary with lactate; EXE+VEH, exercise without lactate; EXE+LAC, exercise with lactate; FNDC5, fibronectin type Ⅲ domain-containing protein 5; BDNF, brain derived neurotrophic factor.

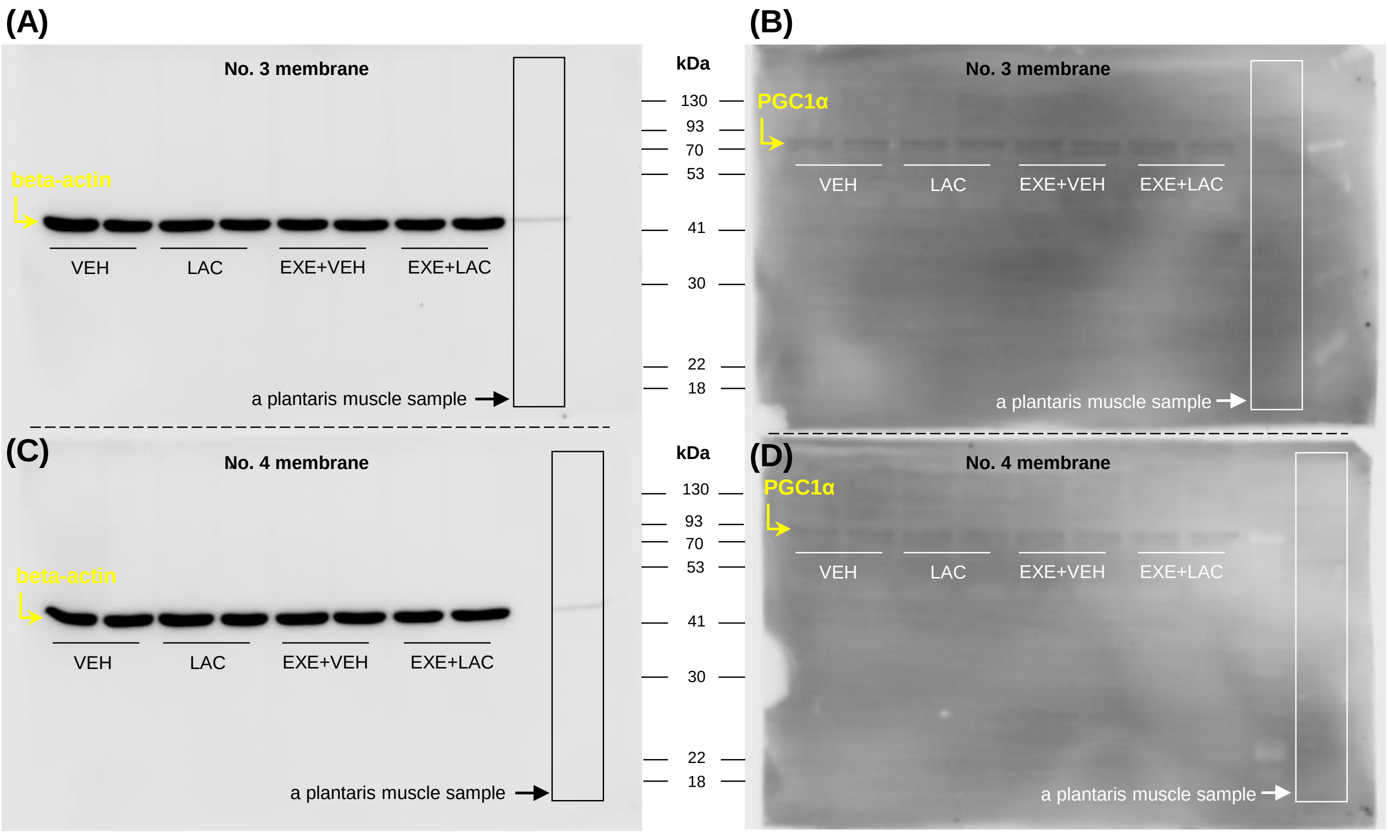

Supplementary Figure 2. The original blots of hippocampal protein expression of PGC1α that presented in Fig. 5D. The result of **(A)** beta-actin and **(B)** PGC1α is obtained from No. 3 membrane. The result of **(C)** beta-actin and **(D)** PGC1α is obtained from No. 4 membrane. To check analysis conditions of plantaris muscle for another study, one plantaris muscle sample was running together. VEH, sedentary without lactate; LAC, sedentary with lactate; EXE+VEH, exercise without lactate; EXE+LAC, exercise with lactate; PGC1α, peroxisome proliferator-activated receptor gamma coactivator 1-alpha.

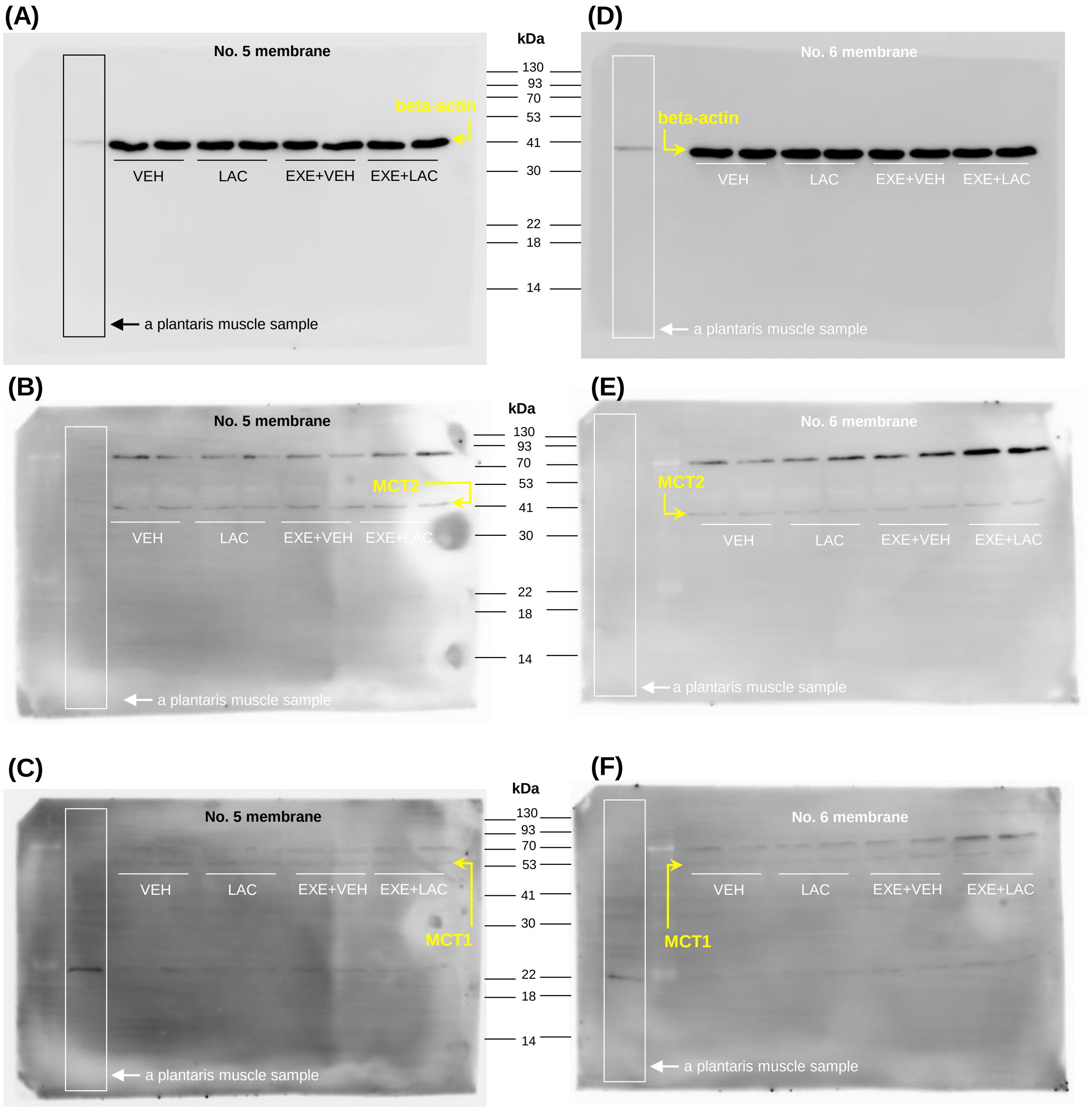

Supplementary Figure 3. The original blots of hippocampal protein expression of MCT2/1 that presented in Fig. 6C. The result of **(A)** beta-actin, **(B)** MCT2, and **(C)** MCT1 is obtained from No. 5 membrane. The result of **(D)** beta-actin, **(E)** MCT2, and **(F)** MCT1 is obtained from No. 6 membrane. To check analysis conditions of plantaris muscle for another study, one plantaris muscle sample was running together. VEH, sedentary without lactate; LAC, sedentary with lactate; EXE+VEH, exercise without lactate; EXE+LAC, exercise with lactate; MCT1/2, monocarboxylate transporter 1/2.

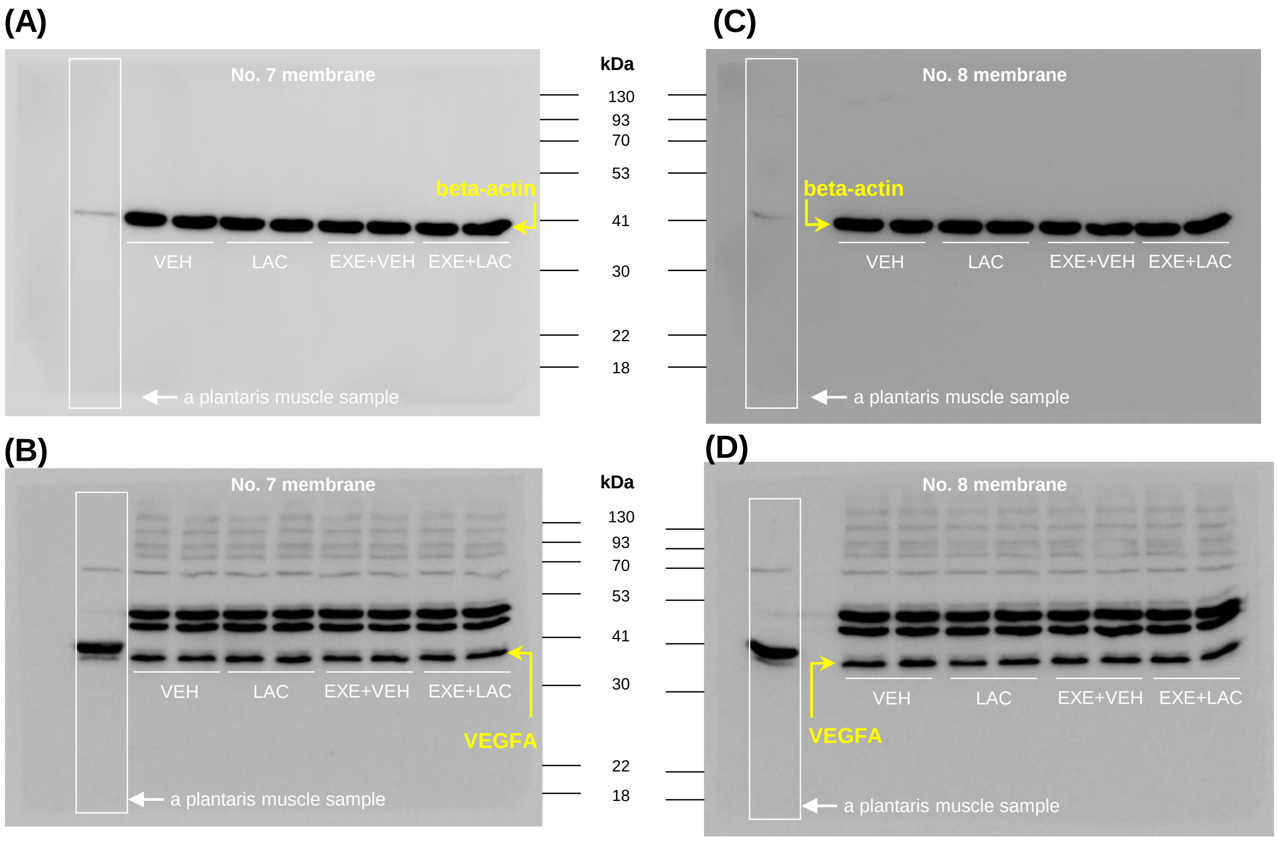

Supplementary Figure 4. The original blots of hippocampal protein expression of VEGFA that presented in Fig. 7C. The result of **(A)** beta-actin and **(B)** VEGFA is obtained from No. 7 membrane. The result of **(C)** beta-actin and **(D)** VEGFA is obtained from No. 8 membrane. To check analysis conditions of plantaris muscle for another study, one plantaris muscle sample was running together. VEH, sedentary without lactate; LAC, sedentary with lactate; EXE+VEH, exercise without lactate; EXE+LAC, exercise with lactate; VEFGA, vascular endothelial growth factor A.

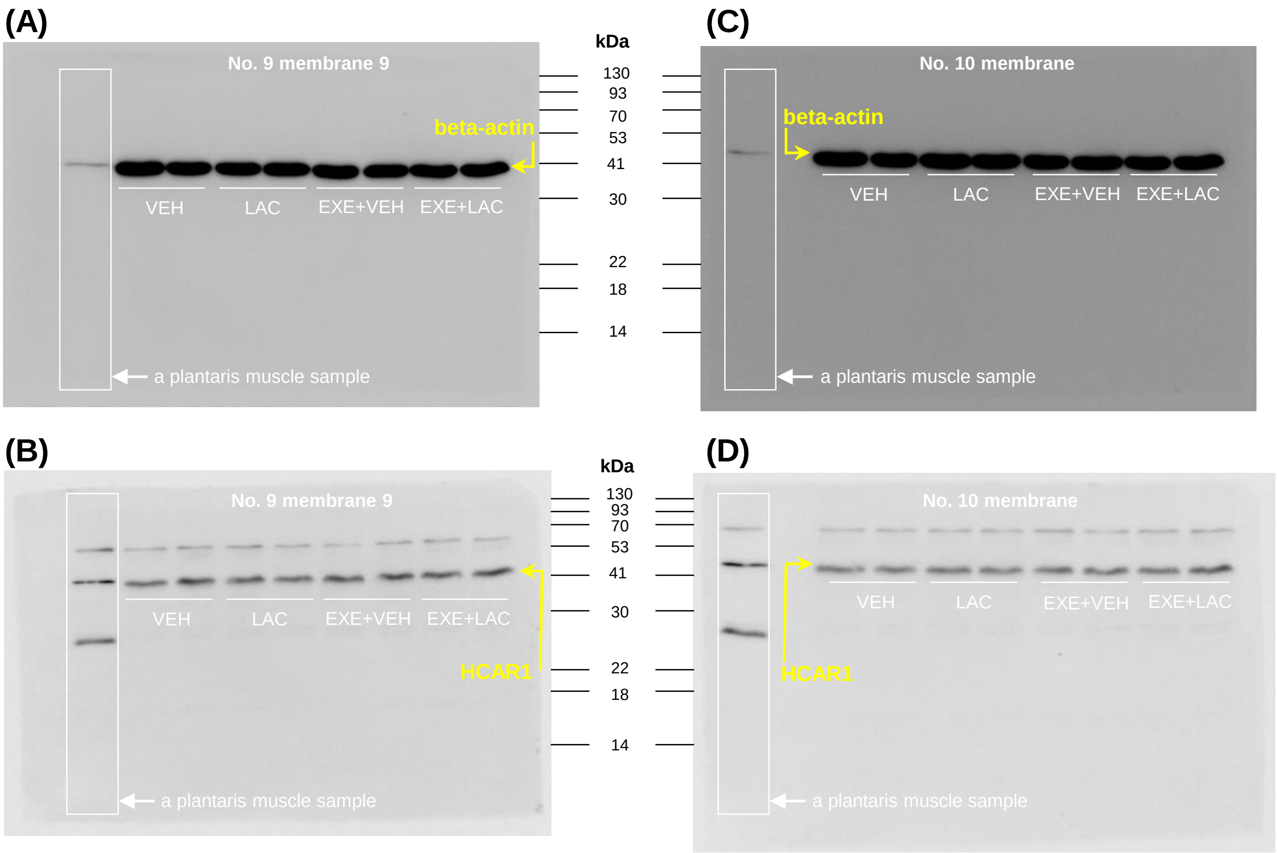

Supplementary Figure 5. The original blots of hippocampal protein expression of HCAR1 that presented in Fig. 7C. The result of **(A)** beta-actin and **(B)** HCAR1 is obtained from No.9 membrane. The result of **(C)** beta-actin and **(D)** HCAR1 is obtained from No. 10 membrane. To check analysis conditions of plantaris muscle for another study, one plantaris muscle sample was running together. VEH, sedentary without lactate; LAC, sedentary with lactate; EXE+VEH, exercise without lactate; EXE+LAC, exercise with lactate; HCAR1, hydroxycarboxylic acid receptor 1.
